# Exogenous Application of Brassinosteroid 24-Norcholane 22(*S*)-23-Dihydroxy Type Analogs to Enhance Water Deficit Stress Tolerance in *Arabidopsis thaliana*

**DOI:** 10.3390/ijms22031158

**Published:** 2021-01-25

**Authors:** Katy Díaz, Luis Espinoza, Rodrigo Carvajal, Evelyn Silva-Moreno, Andrés F. Olea, Julia Rubio

**Affiliations:** 1 Departamento de Química, Universidad Técnica Federico Santa María, Avenida España 1680, Valparaíso 2340000, Chile; katy.diaz@usm.cl (K.D.); luis.espinozac@usm.cl (L.E.); rodrigo.carvajal@postgrado.usm.cl (R.C.); 2 Instituto de Investigación Agropecuarias, INIA–La Platina, Avda. Santa Rosa, Santiago 11610, Chile; evelyn.silva@inia.cl; 3 Instituto de Ciencias Químicas Aplicadas, Facultad de Ingeniería, Universidad Autónoma de Chile, Santiago 8910339, Chile; 4 Instituto de Ciencias Biomédicas, Facultad de Ciencias de la Salud, Universidad Autónoma de Chile, Santiago 8910339, Chile

**Keywords:** phytohormone, brassinosteroids, synthetic analogs, abiotic stress, *Arabidopsis thaliana*, gene expression, real time PCR

## Abstract

Brassinosteroids (BRs) are plant hormones that play an essential role in plant development and have the ability to protect plants against various environmental stresses, such as low and high temperature, drought, heat, salinity, heavy metal toxicity, and pesticides. Mitigation of stress effects are produced through independent mechanisms or by interaction with other important phytohormones. However, there are few studies in which this property has been reported for BRs analogs. Thus, in this work, the enhancement of drought stress tolerance of *A. thaliana* was assessed for a series of 2-deoxybrassinosteroid analogs. In addition, the growth-promoting activity in the Rice Lamina Inclination Test (RLIT) was also evaluated. The results show that analog 1 exhibits similar growth activity as brassinolide (BL; used as positive control) in the RLIT bioassay. Interestingly, both compounds increase their activities by a factor of 1.2–1.5 when they are incorporated to polymer micelles formed by Pluronic F-127. On the other hand, tolerance to water deficit stress of *Arabidopsis thaliana* seedlings was evaluated by determining survival rate and dry weight of seedlings after the recovery period. In both cases, the effect of analog 1 is higher than that exhibited by BL. Additionally, the expression of a subset of drought stress marker genes was evaluated in presence and absence of exogenous applied BRs. Results obtained by qRT-PCR analysis, indicate that transcriptional changes of At*DREBD2*A and *AtNCED3* genes were more significant in *A. thaliana* treated with analog 1 in homogeneous solution than in that treated with BL. These changes suggest the activation of alternative pathway in response to water stress deficit. Thus, exogenous application of BRs synthetic analogs could be a potential tool for improvement of crop production under stress conditions.

## 1. Introduction

The combination of drought and increasing temperature causes extensive agricultural losses, negatively impacting crop production worldwide [1]. To survive under water deficit conditions, plants adapt themselves giving various physiological and biochemical responses to drought stress, with stomata closure being one of the most known physiological plant response to water deficit [2,3,4]. Transduction of different signals lead to expression of genes which code for different proteins accomplishing a variety of functions, such as reducing cell growth, synthesizing abscisic acid (ABA), scavenging ROS, delaying senescence, and enhancing photosynthetic activity [5]. Tolerance to draught stress in *Arabidopsis* is mediated by changes in gene expression [6,7,8], and it has been shown that ~1700 *Arabidopsis* genes exhibit at least a twofold increase in their expression level [9].

Thus, tolerance to water stress deficit has become of the upmost importance due to the climate change that is increasing the extension of arid and semi-arid regions. In this context, it is necessary to create new productive alternatives or find the way to enhance stress tolerance in the natural resources used to feed the growing population.

**Figure 1:**
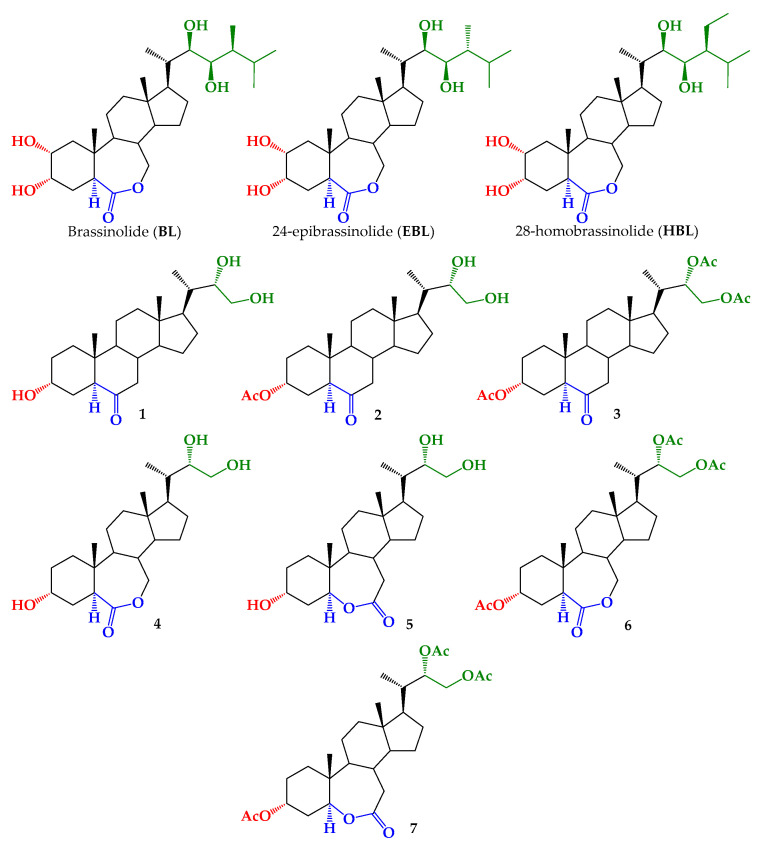
Chemical structures of brassinolide (BL), 24-epibrassinolide (EBL), 28-homobrassinolide (HBL), and BRs 24-nor-5α-cholan type analogs analyzed in this study (1–7).

Brassinosteroids (BRs) are a group of phytohormones that regulates a wide range of biological processes, such as plant growth regulation [10,11], cell division, and differentiation in young tissues of growing plants [12,13,14]. Besides, BRs increase resistance in plants to various kinds of biotic and abiotic stress factors, i.e., low and high temperature, drought, heat, salinity, heavy metal toxicity, and pesticides [15,16,17,18,19,20]. Plants respond and adapt themselves to drought stress in order to survive under water deficit conditions. Drought stress induces various biochemical and physiological responses in plants, being stomata closure the best-known physiological plant response to water deficit [2,3,4]. On the other hand, exogenous application of natural BRs has been used to improve stress tolerance in a variety of plants [21,22]. Brassinolide (BL), 24-epibrassinolide (EBL), and 28-homobrassinolide (HBL) (Figure 1) have been used to assess the effect of biologically active BRs in drought-stressed plants [23]. Most of the published data are related to crop plants, such as wheat [24,25,26], rice [27], sorghum [28], maize [29,30], tomato [31,32,33], and mustard [34], and in general it has been shown that BRs increase antioxidant enzymatic activity and reduce drought stress effects on plants as measured by different biochemical and physiological parameters, i.e., chlorophyll accumulation, activity of antioxidant enzymes, total protein contents, stomatal conductance, photosynthesis, and membrane stability. In leaves of soybean plants, BL increases photosynthesis, the content of soluble sugars and proline, and activities of antioxidant enzymes [35]. Moreover, it was found that EBL was relatively more effective than HBL [27,36].

Despite these studies, the mechanisms by which BRs induce tolerance to water stress have not been fully clarified [37]. It has been shown that BRs produce anti-stress effects independently or by interaction and crosstalk with other plant growth regulators involved in responses to abiotic and biotic stresses, namely, abscisic acid, ethylene (ET), jasmonic acid (JA), and salicylic acid (SA) [38,39,40]. This interaction can occur at the level of signal transduction or gene expression. It has also been shown that enhanced plant tolerance induced by BRs is associated to overexpression of stress responsive genes. Major BR signaling components have been identified for the model plant *Arabidopsis thaliana* [41,42]. Briefly, the current model for BR signal transduction and regulatory mechanisms in response to environmental changes is the following. BRs are perceived by membrane localized serine/threonine receptor-like kinase complex formed by BRASSINOSTEROID-INSENSITIVE 1 (BRI1) and BRI1-ASSOCIATED KINASE 1 (BAK1). BRs perception leads to activation of BRI1 and activated BRI1 initiates a signal transduction cascade resulting in accumulation of two key transcription factors (TFs) known as BRASSINAZOLE RESISTANT1 (BZR1) and BRI1-EMS-SUPPRESSOR 1 (BES1). BZR1 and BES1 can directly regulate the expression of thousands of downstream responsive genes and other transcription factors, determining plant growth and stress tolerance [3,43,44,45].

Taken together, these findings suggest that BRs not only alleviated the antagonistic effect of drought stress, but also enhanced plant growth and yield. Therefore, deciphering the molecular actors in these interactions between BRs analogs and their adaptation to stress is very important to improve the performance efficiency of plants. Exogenous BRs have been applied by seed soaking and/or leaf spraying at concentrations in the range of 10^−6^ to 10^−9^ M over a great variety of plants [29,35,46,47]. However, just a few works have used synthetic BRs analogs [48,49,50]. Much effort has been dedicated to synthesizing these compounds, mainly by the extremely low quantity of BRs existing in plants and their extremely high cost, which make its use prohibitive in agriculture applications. Currently, natural molecules with molecular structures similar to BRs are being used to synthesize numerous analogs with structural changes on the A/B rings and/or on the side chain have been reported [12,51]. Some BR analogs with drastic structural modifications in the side chain have also shown activities as plant growth regulators [52,53,54]. Thus, it would be interesting to determine the drought stress tolerance induced by BR analogs exhibiting growth enhancing activity. Therefore, to address this point we have used previously synthesized BRs 24-nor-5α-cholan type analogs (Figure 1, 1–7) [55,56].

The growth-promoting activity of BRs analogs 1–7 (Figure 1) and its protective effect against water stress in *Arabidopsis thaliana* plants by expression of associated genes have been evaluated in aqueous solution and polymeric micelles. Several mechanisms have been proposed to explain how endogenous BRs mediate the adaptation of plants to different abiotic stresses. Some of them are independent and other involves interaction and crosstalk of BRs with ABA and other plant growth regulators. ABA is a key signaling pathway involved in responses to abiotic and biotic stresses that regulate expression of many stress responsive genes. Therefore, through this study we intend to detect and identify any alteration on the transcription of genes, involved in water stress caused by water deprivation, induced by exogenous BRs analogs. These data would allow us to throw some light upon the mechanism by which exogenous analog improves the tolerance of plants against abiotic stress.

## 2. Results and Discussion

The synthesis and characterization of all BRs analogs used in this work have been reported previously. Briefly, brassinosteroids 24-nor-5α-cholan type analogs (1, 2) have been synthesized by dihydroxylation of a terminal olefin (3α-Acetoxy-24-nor-5α-cholan-22-en-6-one) obtained from hyodeoxycholic acid in a six steps route [56]. Baeyer-Villiger reaction of analog 3, leads to lactones 6 and 7, which by mild deacetylation produces 4 and 5 [55].

### 2.1. Growth-Promoting Activity of BRs Analogs in Aqueous Solution and Polymeric Micelles (Pluronic F-127) in the RICE Lamina Inclination Assay

**Figure 2:**
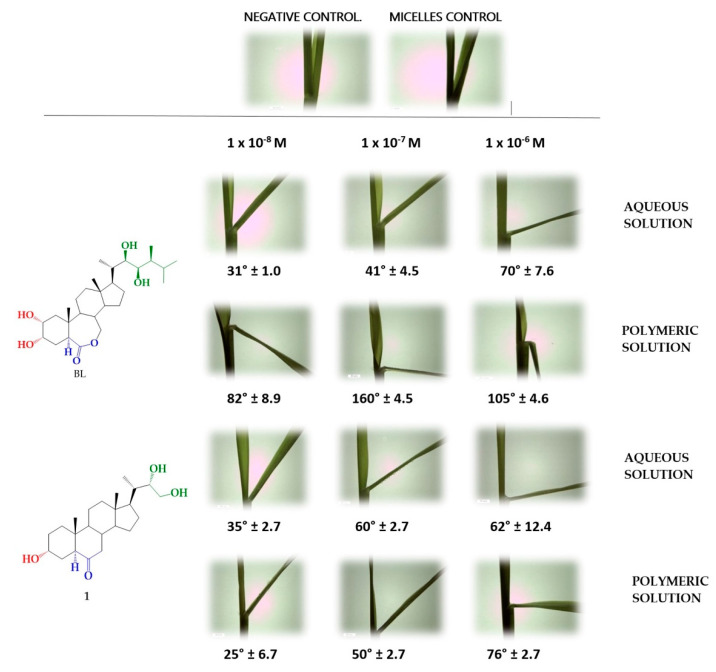
RLIT for analog 1, compared to aqueous or polymer solution (negative control) and BL in presence or absence of Pluronic F 127 1 mM (positive control). Both 1 and BL were assayed at concentration ranging from 1 × 10^−8^ to 1 × 10^−6^ in aqueous and polymer solutions.

The activity of analogs 1–7 was evaluated using the Rice Lamina Inclination Test (RLIT). In Figure 2 are shown visual images of the degree of inclination of the rice lamina (*Oryza sativa* cultivar Zafiro) obtained in presence of BRs analog (1) and BL, which was used as positive activity control.

The results are collected in Table 1 and correspond to the average opening angle ± DS, product of six independent replicas for concentrations 1 × 10^−8^ M, 1 × 10^−7^ M, and 1 × 10^−6^ M measured in aqueous solution and in presence of 1 mM of Pluronic F 127.

The data in Table 1 show that BL is the most active growth promoter in the whole range of tested concentrations. On the other hand, analogs 1, 3, 4, and 6 applied in aqueous solution induce similar or slightly lowest bending angles, whereas analogs 2, 5, and 7 are much less active at the three tested concentrations. For all BRs analogs there is a direct dependence of growth-promoting effect with BRs analogs concentration. This is valid for all BRs analogs except 1 and 3, which exhibit abnormally high values at 1 × 10^−7^ M.

To correlate the measured activity with the chemical structure of BRs analogs we must differentiate them according to hydroxylation (1, 2, 4, 5) or acetylation (3, 6, 7) of the C22-C23 carbons in the 24-nor side chain, and the presence and orientation of the lactone function in the B ring (4–7). Comparison of data for analogs 1 and 3, 4 and 6, and 5 and 7 suggests that di-acetylation of -OH groups in the side alkyl chain has no effect on RLIT activity. However, acetylation of just one hydroxyl group in the ring A produces a drastic reduction on it (compare 1 and 2). In previous work, it has been shown that the growth-promoting effect is not affected by changing one -OH by a benzoyl group. Results obtained in a molecular docking study suggest that the alkyl side chain in BL contributes significantly to recognition through the interactions of the hydrophobic end with BAK1 [57]. Thus, high promoting activities of BRs analogs with shortest side chain can be explained by the presence of slightly bulky and hydrophobic substituent in the chain end. These results are in line with previous work where it has been proposed that activity of synthetic analogs is not determined by the presence or absence of a chemical group, but instead depends on the oxygen atoms spatial distribution [58].

The effect of polymeric micelles on BRs analogs activity depends on the chemical structure as well. Thus, BL and analog 2 incorporated to polymer micelles of Pluronic F 127 increase their activity by a factor ranging from 1.5 to 3.9 (see Figure 2 and Table 1). On the other hand, polymer micelles decrease the activity of analogs 1 and 3 at the lower concentrations, whereas exert no effect on activity of analog 6. These effects must be related to changes on bioavailability due to solubilization into polymer micelles. Under these experimental conditions, i.e., high concentration ratio of polymer to BRs analogs, the occupation number (number of solute molecules by micelle) is very low and the solubilization process is described by a pseudo-phase equilibrium [59]. In this model, the transfer from the aqueous phase to the micelle is measured by the partition coefficient, and free energy of transfer can be obtained from this data. Several relationships between free energy and chemical structures have been established [60,61,62], and the main factors favoring the transfer of a solute from the aqueous phase to the micelle are the increase of the total solute hydrophobicity and change in the relative positions of hydrophilic groups from *para* to adjacent positions. The latter term is due to interaction between solute and the micelle microenvironment [63]. The structures of the BL and BRs analogs are very similar and clearly all these compounds are hydrophobic. Therefore, in the presence of polymer micelles these molecules will be incorporated into the non-polar microdomain provided by the micelle core. The location of molecules into these nanoaggregates is determined by specific interactions between the molecule and the core-forming polymer. Thus, analogs 1 and 2 will be located with the -OH groups of the alkyl chain orientated to the more polar surface. However, analog 1 has an additional -OH group in C3 that anchor the molecule by forming hydrogen bond with the EO groups existing in the micelle core. This interaction is not present in analog 2 and consequently the exit of this molecule is much easier, and its activity is increased. The total absence of -OH groups in analog 3 bury this molecule into the micelle core, reducing its availability and its activity. Thus, these results suggest that to achieve better solubility and bioavailability it is necessary to consider the structural factors controlling their solubility and interaction with the nanoaggregate.

### 2.2. Effect of BRs on Water Deprivation Tolerance in Arabidopsis Thaliana Seedlings

The main aim of this work was to evaluate the anti-stress tolerance induced by BRs analogs exhibiting growth-enhancing activity. Therefore, this study was carried out using analog 1, which turned out to be the most active growth promoter both in aqueous and polymer micellar solutions.

**Figure 3:**
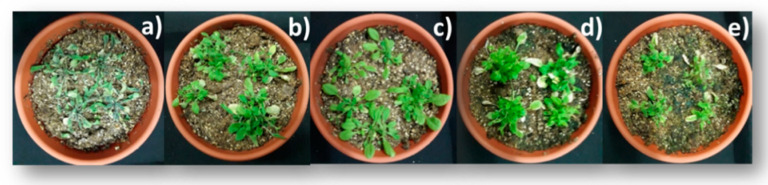
Effect of BRs and biostimulant on water deficit stress tolerance of *A. thaliana* seedlings: (**a**) non-treated samples (negative control), (**b**) analog 1, (**c**) NAP, (**d**) 1 incorporated in polymer micelles (1E), and (**e**) BL. BL, 1, and 1E were applied at concentration of 1 × 10^−7^ M; Pluronic F 127 1 mM. The photographs were taken 2 days after the drought process was initiated.

*Arabidopsis thaliana* seedlings, closed under different treatments, were subjected to water deprivation conditions for five days. The surviving seedlings were then regularly watered once a day (20 mL for pot) and left to grow in a recovery process lasting two days. Some visible morphological parameters, such as wilting of the leaves, decrease in growth, and complete dryness of plants, were used to monitor plant changes caused by water stress in presence and absence of BRs. Besides, a commercial natural biostimulant (Nutrafol Amino Plus; NFP) formulated mainly as a mixture of amino acids and polypeptides, was applied for comparison. Results obtained two days after water deprivation are shown in Figure 3.

It is clearly observed that all applied treatments enhance the tolerance of *A. thaliana* to water deficit following the order: BL = NAP > 1 > 1(E).

**Figure 4:**
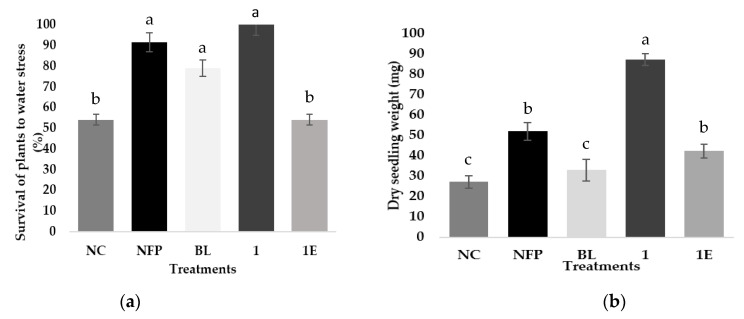
Effect of BRs analogs on water-stress tolerance in *Arabidopsis thaliana*. (**a**) Proportion of plants recovered two days after ending of water deprivation period; (**b**) Dry weight of surviving plants. NC, NFP, BL, 1, and 1E denote non-treated samples, Nutrafol AminoPlus, brassinolide, analog 1 in aqueous solution, and analog 1 in polymer micelles, respectively. Data were obtained using 40 plants/treatment. Different upper letters (a, b, c) indicates that those values are statistically different (*p* < 0.05), whereas identical letters means that values are the same. Post hoc Tukey HSD test was applied to show statistically significant differences among the means.

At the end of this bioassay, plants were dried at 80 °C for 24 h and the survival rate and the dry weight were evaluated. The results are shown in Figure 4.

The data shown in Figure 4a indicate that only half of the plants were able to survive to draught stress conditions (NC). Similar survival rate was obtained for plants treated with 1 dissolved in polymer micelles (1E). On the other hand, 100% of plants treated with analog 1 remained alive, exceeding by 10% and 20% the ratio reached by treatment with NFP and BL, respectively. During the recovery phase, all surviving plants reassumed their development and growing. The effect of treatments in this stage is evaluated by the average dry weight of plants, and these results are shown in Figure 4b. The lowest dry weight (27 mg) corresponds to non-treated plants (NC), indicating that draught stress affects negatively plant growing. Surprisingly, BL and 1E exhibit a very small growth promoting effect, i.e., 30 and 40 mg, respectively, whereas 1 applied in aqueous solution induces a growth enhancement that is 2–3 times larger. It has been shown that exogenous application of BRs increases chlorophyll content and photosynthesis in plants under stress [25,29,32,46]. These are key factors for growing and therefore are closely related to the outcome of dry weight measurements. In general, results obtained in both measurements indicate that application of exogenous BRs increases the tolerance of *A. thaliana* to water deficit stress. These results are in line with a study of the effect of exogenous application of EBL on Basmati rice submitted to salinity stress [64]. Besides, analog 1, which is the more efficient exogenous anti-stress molecule used in this work, has also demonstrated interesting effects when is applied to grapes and berries, i.e., increasing of index color values by increasing the content of soluble solids and dehydroxylated anthocyanins [65]. Thus, exogenous application of BRs analogs is a promising tool for enhancement of growth and stress tolerance in plants.

Several mechanisms have been proposed to explain how endogenous BRs mediate adaptation of plants to different abiotic stresses. Some of them are independent and other involves interaction and crosstalk of BRs with ABA and other plant growth regulators. ABA is a key signaling pathway involved in responses to abiotic and biotic stresses that regulate expression of many stress responsive genes. Therefore, through this study we intend to detect and identify any alteration on the transcription of genes, involved in water stress caused by water deprivation, induced by exogenous BRs analogs. These data would allow us to shed some light upon the mechanism by which exogenous analog improves the tolerance of plants against abiotic stress.

### 2.3. Relative Expression of Drought Stress Induced Genes

ABA is a key signaling pathway involved in responses to abiotic and biotic stresses that regulate expression of many stress responsive genes, and is quickly accumulated by expression of 9-cis-epoxycarotenoid dioxygenase (NCED) gene, *AtNCED3*, in response to draught stress [6]. Subsequently, ABA mediates the expression of stress-related genes such as *rd22* (responsive to desiccation 22) in *A. thaliana* [66,67].

On the other hand, a very important ABA-independent signal transduction pathway involves the cis-acting element, dehydration response element (DRE) *AtDREB2A*, a transcriptional factor that recognizes DRE activating the expression of downstream stress-responsive genes in *Arabidopsis thaliana* [7,8].

Finally, BRs signaling pathway under drought stress might be conditioned by BRs accumulation. 3-Hydroxy-3-methylglutaryl-CoA reductase 1 should be involved in this process (HMG1), it is a key limiting enzyme that catalyzes the synthesis of mevalonate, a precursor in the isoprenoid biosynthetic pathway leading to phytosterols. Campesterol, one of these sterols, is the precursor of BR biosynthesis and therefore any change in BRs content should be reflected by an increase on *AtHMG1* activity [68].

Thus, in this work, the expression of these genes in absence (NC) and presence of BRs (BL, 1, 1E) have been analyzed at two different times, namely, five days after the initiation of water deprivation period (T_1_) and two days after watering in the recovery period (T_2_).

**Figure 5:**
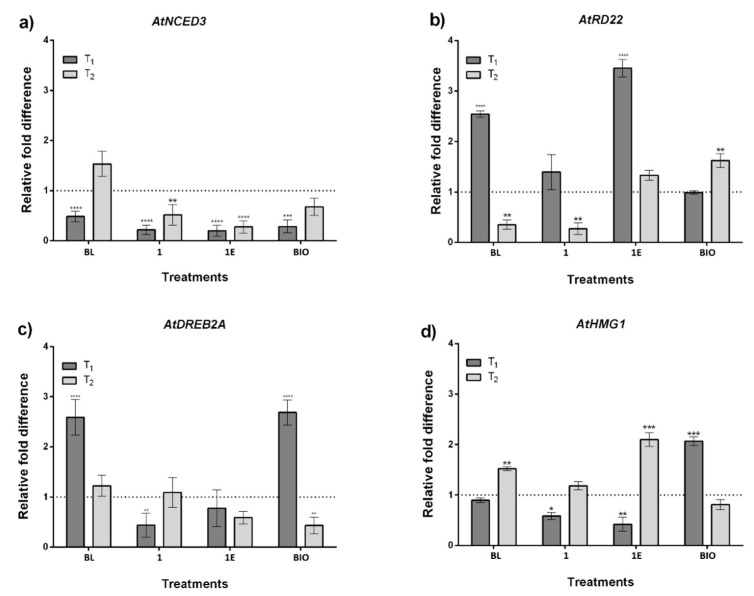
Transcript expression of drought stress responsive genes in *A. thaliana* at different times after application of BRs (BL, 1, 1E) and Biostimulant (NFP). T_1_ correspond to five days after beginning of water deprivation period, and T_2_ is two days after watering in the recovery period. Relative transcript expression levels of (**a**) *AtNCED3*, (**b**) *AtRD22*, (**c**) *AtDREB2A*, and (**d**) *AtHMG1* were analyzed by qRT-PCR. Dashed line represents expression level of non-treated samples. Data represent the means of three independent repeats replicates ± SD (*n* = 9), asterisks indicate significant differences compared with negative control under the same treatment conditions: * *p* < 0.05, ** *p* < 0.01, *** *p* < 0.001, **** *p* < 0.0001. The significance of difference was analyzed by Dunnett’s multiple comparisons test (*p* < 0.05).

Changes in gene expression were analyzed using quantitative real-time PCR (qRT-PCR), which allows one to determine changes in the expression (overexpression or inhibition) of the genes of interest, relative to the expression of an endogenous gene. Fold changes in transcript accumulation in treated samples relative to NC were calculated using 2^−ΔΔ*C*t^ method [69]. The obtained results are shown in Figure 5.

The expression of the *AtNCED3* gene is shown in Figure 5a, it is involved in the accumulation of ABA, and the expression of *AtRD22* is shown in Figure 5b, a drought-responsive gene mediated by ABA. It has been reported that plants subjected to draught stress exhibit 1.7 to 17-fold increase in *AtNCED3* gene expression depending on the drought level [70]. According to the mathematical analysis of ΔΔ*C*t used in this work, this level is indicated by the dashed line at unity value of relative fold difference. Interestingly, in Figure 5a it can be seen that the presence of BL, 1, and 1E downregulate *AtNCED3* after five days of water stress (T_1_). The relationship between morpho-physiological responses and expression levels of draught-responsive genes has recently been established [71]. Specifically, it has been shown that the leaf dry matter content decreases with increasing level of *AtNCED33*. Our results indicate that plants treated with exogenous BRs showed the lowest expression values of *AtNCED3* and the highest dry seedling weights (see Figure 4b), and that only in the presence of BL its level is 1.5-fold overexpressed after two days of recovery (T_2_). Thus, these results are in line with the proposed relationship. Despite of the BRs-induced decreasing of *AtNCED3* and consequently a lowest level of ABA accumulation [72], the expression of *AtRD22* is upregulated by all BRs at T_1_. Expression of this gene is necessary for drought tolerance in plants, and therefore these results suggest that exogenous applied BRs provide an alternative ABA-independent pathway for expression of *AtRD22* in response to water deficit stress. In a possible parallel pathway, *AtRD22* expression can be modulated by MYC, a transcription factor that has been described as a regulator of ABA and JA signaling pathways [73].

After alleviating of stress conditions (T_2_) the expression of these genes is still down regulated or reach the level of non-treated plants (BL, *AtNCED3*; 1E, *AtRD22*).

In the ABA-independent signaling pathway, BL overexpress the ABRE transcription factor activation, *AtDREB2A*, by a factor of three, whereas 1 and 1E show a slightly downregulation effect on this gene expression. Thus, BL upregulate the expression of *AtRD22* and *AtDREB2A* under water deprivation conditions. At T_2_, all treatments group reach relative expression level similar to the NC (Figure 5c). There is a complex crosstalk between ABA-dependent and ABA-independent pathways.

Finally, in Figure 5d is shown the expression of *AtHMG1*, a gene that is involved in the synthesis of phytosterols, which regulate membrane fluidity [74]. It has been reported that *AtHMG1* activity increases with increasing severity of drought stress, which leads to higher levels of phytosterols [75]. However, our results indicate that at T_1_, *AtHMG1* is downregulated in plants treated with 1 and 1E, whereas *AtHMG1* expression of plants treated with BL is similar to that of NC group. This behavior could be attributed to the effect of exogenous BRs application on BRs biosynthesis. It has been reported that several BR biosynthetic genes are downregulated by exogenous BL application [68]. Our results are in line with this behavior, namely, there are no significant *AtHMG1* expression changes during water deficit period and only in the recovery phase it is possible to observe a 1.5-to-2-fold difference in the *AtHMG1* expression in plants treated with BL and 1E, whereas 1 and NFP showed the same expression of control group. The upregulated expression observed with BL and 1E treatments, could be related with metabolic recovery activity of mevalonate and BRs precursors (Figure 5d).

Thus, our results show that exogenous application of BL and the synthetic analog 1 results in improvement of the morphophysiological response of *A. thaliana* to draught stress. This effect has been observed for natural BRs on different crops and attributed to crosstalk with other plant regulators. For example, it has been shown that EBL enhances plant adaptation to abiotic stress conditions by modulation of *A. thaliana* stomatal closure via ethylene signal transduction pathway, Gα protein activation, hydrogen peroxide, and nitric oxide cascade production [76]. Interestingly, our results indicate that BL and 1 induce overexpression of *AtRD22*, with no participation of *AtNCED3* or ABA accumulation, which is related with stomatal closure mechanism. Thus, it seems that synthetic analog 1 might also participate in these alternative ways of gene expression, and therefore it would be worth trying to establish a relationship between this activity and chemical structure of BRs analogs.

## 3. Materials and Methods

### 3.1. Preparation of Aqueous and Polymeric Solution of Tested Compounds

The main drawback in the application of exogenous BRs analogs is their poor water solubility, and therefore it is not possible to determine the exact quantity of BRs entering into the plant. To overcome this problem, we have used polymer micelles to increase their bioavailability. These micelles represent only one type of many nanostructures that are being developed for application of crop protecting agents [77,78]. Most of polymer micelles are spherically shaped core–shell structures where the hydrophobic segments of an amphiphilic polymer form the core of the micelle while the hydrophilic parts form the corona or outer shell. The hydrophobic micelle core provides a nonpolar microenvironment where lipophilic compounds could be incorporated, whereas the hydrophilic shell helps to avoid micelle aggregation and to ensure micelle solubility. In this study, we have used a commercial triblock copolymer, poly(ethylene glycol)-poly(propylene glycol)-poly(ethylene glycol), available as Pluronic F-127, which we have used before to increase antifungal activity [79].

To determine the biological activity of BRs analogs and the positive control BL, homogeneous stock solutions were prepared by dissolving 10 mg of each compound in ethanol (1 mL) and then diluted in water to obtain a concentration of 1 × 10^−5^ M. Different aliquots of these solutions were added to reach final concentrations ranging between 1 × 10^−8^ and 1 × 10^−6^ M.

Polymer micelles were prepared by direct dissolution of Pluronic F 127 (Aldrich) in water at a concentration well above the critical micelle concentration (1 × 10^−3^ M) [62,79]. BRs analogs were incorporated in polymer micelles by using the emulsion method [80].

### 3.2. Biological Activity: Rice Lamina Inclination Assay

The growth promoting activity of compounds 1–7 (Figure 1) was evaluated by the Rice Lamina Inclination Test [81,82] according to a described procedure [54]. Seeds of rice (*Oryza sativa*) Zafiro variety, provided by the Institute of Agricultural Research (INIA-Quilamapu-Chile), were used. Six segments per treatment were incubated in a Petri dish containing 60 mL of distilled water and finite quantities of BRs analogs 1–7, in aqueous solution or incorporated in polymer micelles, were added to reach final concentrations 1 × 10^−8^ M, 1 × 10^−7^ M, and 1 × 10^−6^ M. BL was used as positive control at the same concentrations, whereas two negative controls were prepared, one that only has sterile distilled water and another which contains micelle solution (Pluronic F-127 1 × 10^−3^ M). This experiment was made twice using six segments each time, so the bending angle values were calculated using twelve segments for each treatment. Finally, the magnitude of the angle induced between the blade and the sheath was measured. Images were taken using a Leica EZ4HD Stereo Microscope with camera software.

### 3.3. Effect of Analog 1 and BL on Water Stress Tolerance of Arabidopsis thaliana

#### 3.3.1. Plant Material and Growth Conditions

Sterilized seeds of the ecotype Columbia wild type (Col-0) of *Arabidopsis thaliana* (obtained from ARABIDOPSIS BIOLOGICAL RESOURCE CENTER (ABRC)) were sown in Petri plates on nutrient culture medium [83] with vitamins, 1% sucrose, and solidified with 1% of agar. Seeds were stratified (4 °C) in the dark for 3 days prior to the germination process. Subsequently the plates were kept at 22 °C, 50% humidity, under photoperiods 16 h light (80 µEm^−2^s^−1^) and 8 h dark.

#### 3.3.2. Water Stress Treatments

After one month of growth in in vitro culture chamber, 40 plants by treatment (4 by pot) were transferred into pots containing sterile substrate-vermiculite (Figure S1a). After one week of adaptation and reestablishment in growth, BL and BRs analog 1, in its aqueous and encapsulated forms (1E), were applied at a final concentration of 1 × 10^−7^ M. Commercial Nutrafol Amino Plus (FERPAC) was applied to the same concentration used on field (200 cc/Hl). Addition of pure water to seedlings was considered as non-treated samples (NC). Subsequently, plants were subjected to water deprivation process for five days (Figure S1b). The soil water potential was measured daily using ProCheck with MPS-6 sensor (Decagon Devices Inc., Pullman, WA, USA) to estimate the level of drought stress (the average soil water potential was below −0.03 MPa) (Figure S1c). Stress conditions remained until the plants showed visible wilt symptoms. Then, surviving seedlings were watered continuously for a two day recovery period. During this time, the soil water content was kept at more than 80% of field capacity. Plants that survived and continued growing were manually counted and their dry weight was quantified (Figure S1g,h). The value of average percentage was determined using 40 seedlings of *A. thaliana*. (two-sided chi-squared test, *p*-value < 0.01). This experiment was repeated twice, and for comparison all plants were kept under the same growth conditions, temperature, light, and soil conditions inside the growth chamber (22 °C, 50% humidity, photoperiods 16 h light and 8 h dark). The soil water potential of each pot was measured daily as described above and used as an indicator for the level of drought stress.

#### 3.3.3. RNA Isolation and qRT-PCR Analysis

Plant tissue (100 mg) was collected, five days after the initiation of draught period (T_1_) and two days after beginning of recovery period (T_2_), stored in 2 mL storage tubes with RNA Later (Merck, Darmstadt, Germany), and then powdered in liquid nitrogen for RNA extraction (Figure S1d–f).

RNA was extracted using the kit NucleoSpin^®^ RNA plant of MN (Macherey-Nagel, Düren, Germany). Once the quality parameters (1.5% MOPS gel in 1× APR) and RNA quantity (Infinite 200 PRO, Tecan Trading AG, Switzerland) were determined, 1 μg of RNA was used for cDNA synthesis using the GoScript Retro Transcription kit (Promega, WI, USA) following the manufacturer’s instructions. Reactions were performed using master mix KAPA SYBR^®^ FAST qPCR Master Mix (2×) kit (Sigma-Aldrich, MO, USA) in the Lightcycler 96 (Roche, Basel, Switzerland) thermal cycler equipment under conditions suggested by the manufacturer. The qRT-PCR cycles had the following reaction conditions: 95 °C for 3 min, 40 cycles comprising denaturation at 90 °C for 3 s, and annealing and extension at 60 °C for 30 s. A list of 11 primers pairs described as indicators of water stress were selected and tested under previously described conditions (Table S1). Eight primers pairs out of this list were suitable for our purposes, but only four pairs had the same efficiency (E = 2): *DREB2A*, *HMG1* [9], *AtNCED3* [70] and RD22 [84]. Cycle threshold (Ct) values obtained for ACTIN2 gene were used to normalize data [85]. For all qRT-PCR experiments, two independent biological replicates were included, and reactions were performed in triplicate. Fold change in transcript accumulation in stressed samples relative to non-stressed sample was calculated using 2^−ΔΔ*C*t^ method [69]. For the analysis and treatment of data obtained using 2^−ΔΔ*C*t^ method, the statistical analysis Two-ways ANOVA and Holm–Sidak approach to multiple comparisons were used to determine statistical differences between each treatment and non-treated samples (NC) with the GraphPad PRISM 6 software (V 6.01) (GraphPad Software Inc., San Diego, CA, USA).

## 4. Conclusions

The results of this study are in line with previous works in which exogenous application of natural BRs can be used for the enhancement of stress tolerance in a variety of plants. However, in this study we have shown that similar results can be obtained by using synthetic BRs analogs with 24-norcholane 22(S)-23-dihydroxy structural conformation. Application of these derivatives in aqueous solution or incorporated to polymer micelles formed by Pluronic F 127 induces morphophysiological responses of *A. thaliana* to water deprivation stress. This effect is reflected in the number of surviving plants and their dry weight after the drought period compared to the control. By using qRT-PCR, it was possible to determine relative gene expression changes at two different times, namely, during the stress period and after water deprivation has ended. Changes in *DREB2A* and *AtNCED3* suggest the activation of alternative pathway in response to water stress deficit. In this parallel pathway, the expression of RD22 is modulated by MYC, a transcription factor that has been described as a regulator of BRs synthesis.

Therefore, exogenous application of BRs synthetic analogs is a potential tool for improvement of crop production under stress conditions.

## Figures and Tables

**Table 1:** Effect of analogs of BRs on the rice lamina inclination (angle opening (°)) in aqueous solution and polymeric micelles (Pluronic F 127 1 mM). Chemical structures of BL and analogs 1–7 are shown in Figure 1. NC denotes non-treated samples.

	Bending Angle Between Laminae and Sheaths (Degrees ± Standard Error))					
	Aqueous Solution			Polymeric Micelles (Pluronic F-127)		
BRs Analogs	1 × 10^−8^ M	1 × 10^−7^ M	1 × 10^−6^ M	1 × 10^−8^ M	1 × 10^−7^ M	1 × 10^−6^ M
BL	31 ± 1 ^a^	41 ± 5 ^b^	70 ± 8 ^a^	82 ± 9 ^a^	160 ± 5 ^a^	105 ± 5 ^a^
1	35 ± 3 ^a^	60 ± 3 ^a^	62 ± 12 ^a^	25 ± 7 ^b^	50 ± 3 ^b^	76 ± 3 ^b^
2	5 ± 3 ^b^	13 ± 2 ^d^	28 ± 3 ^b^	16 ± 5 ^b^	52 ± 6 ^b^	43 ± 6 ^c^
3	34 ± 2 ^a^	45 ± 3 ^b^	53 ± 6 ^a^	6 ± 2 ^c^	25 ± 3 ^c^	45 ± 5 ^c^
4	40 ± 3 ^a^	37 ± 4 ^b^	46 ± 3 ^b^	17 ± 2 ^b^	37 ± 2 ^c^	53 ± 4 ^c^
5	18 ± 2 ^b^	24 ± 2 ^c^	48 ± 9 ^b^	-	-	-
6	33 ± 3 ^a^	45 ± 2 ^b^	62 ± 10 ^a^	22 ± 4 ^b^	59 ± 7 ^b^	75 ± 5 ^b^
7	13 ± 4 ^b^	25 ± 5 ^c^	42 ± 4 ^b^	-	-	-
NC	7 ± 5 ^b^	7 ± 5 ^d^	7 ± 5 ^c^	10 ± 3 ^c^	10 ± 3 ^d^	10 ± 3 ^d^

These values represent the mean ± standard deviation of two independent experiments with at least six replicates each (*n* = 12). Different upper letters (a, b, c, d) indicates that those values are statistically different (*p* < 0.05), identical letters means that values are the same. Post hoc Tukey honestly significant difference (HSD) test was applied to show statistically significant differences among the means.

## Data Availability

Data is contained within the article or Supplementary Material.

## Supplementary Materials

Supplementary Materials can be found at https://www.mdpi.com/1422-0067/22/3/1158/s1. Table S1: Reference genes selected by response to deficit water stress in Arabidopsis thaliana. Figure S1: Evaluation process of in vivo bioassay of abiotic stress due to water deprivationF.

## Abbreviations

NC	Non-treated samples
qRT-PCR	Quantitative real-time PCR
NFP	NUTRAFOL AMINO PLUS
