# Supplementary material for: Exogenous Application of Brassinosteroid 24-Norcholane 22(S)-23-Dihydroxy Type Analogs to Enhance Water Deficit Stress Tolerance in Arabidopsis thaliana
Source: Int J Mol Sci. 2021 Jan 25;22(3):1158. doi: 10.3390/ijms22031158 (PMC7865588; doi:10.3390/ijms22031158)
Supplement: Supplementary file 1 [file ijms-22-01158-s001.pdf]

International Journal of *Molecular Sciences*

MDPI

Article

Exogenous Application of Brassinosteroid 24-Norcholane 22(*S*)-23-Dihydroxy Type Analogs to Enhance Water Deficit Stress Tolerance in *Arabidopsis thaliana*

Katy Díaz [1], Luis Espinoza [1], Rodrigo Carvajal [1], Evelyn Silva-Moreno [2], Andrés F. Olea [3,*] and Julia Rubio [4,*]

1 Departamento de Química, Universidad Técnica Federico Santa María, Avenida España 1680, Valparaíso 2340000, Chile; katy.diaz@usm.cl (K.D.); luis.espinozac@usm.cl (L.E.); rodrigo.carvajal@postgrado.usm.cl (R.C.)
2 Instituto de Investigación Agropecuarias, INIA–La Platina, Avda. Santa Rosa, Santiago 11610, Chile; evelyn.silva@inia.cl
3 Instituto de Ciencias Químicas Aplicadas, Facultad de Ingeniería, Universidad Autónoma de Chile, Santiago 8910339, Chile
4 Instituto de Ciencias Biomédicas, Facultad de Ciencias de la Salud, Universidad Autónoma de Chile, Santiago 8910339, Chile
* Correspondence: andres.olea@uautonoma.cl (A.F.O.); julia.rubio@uautonoma.cl (J.R.); Tel.: +56-322-652-843（A.F.O. & J.R.）

Table S1: Reference genes selected by response to deficit water stress in *Arabidopsis thaliana*.

Gene	Gene locus	Name	Response	Related with…	Reference
ACT2_2 *	AT3G18780	ACTIN 2	no changes quantified	Structural constituent of cytoskeleton	[79] Yamashita et al., 2016
DREB2A	AT5G05410	DRE-BINDING PROTEIN 2A	up-regulated	Regulates expression of many genes inducible by water stress	[63] Catala et al., 2007
HMG1	AT1G76490	HYDROXY METHYLGLUTARYL COA REDUCTASE 1	up-regulated	Mevalonate biosynthesis	[63] Catala et al., 2007
NCED3	AT3G14440	NINE-CIS-EPOXYCAROTENOID DIOXYGENASE 3	up-regulated	ABA synthesis in response to drought and salinity stress	[68] Gupta et al., 2016
RD22	AT5G25610	RESPONSIVE TO DESICCATION 22	up-regulated	ABA mediated dehydration response	[78] Kim et al., 2013

* endogenous reference gene

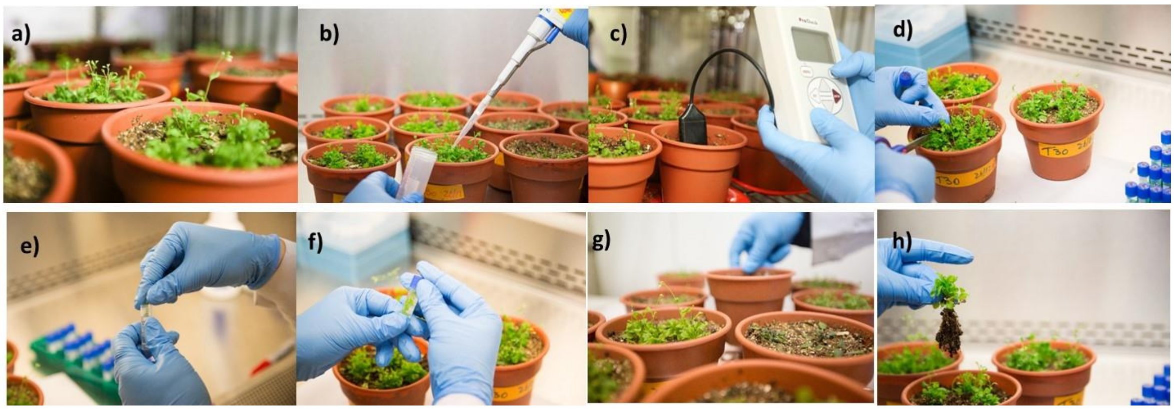

Figure S1. Evaluation process of in vivo bioassay of abiotic stress due to water deprivation: a) Transplant of healthy Arabidopsis thaliana seedlings growing in vitro to pots kept in a plant growing chamber under controlled conditions; b) Application of treatments; c) Monitoring of soil water potential; d) Random plant tissue cutting; e)

and f) Collect and quick-frozen of samples in RNA ladders for qRT-PCR; g) Extraction of seedlings to quantify dry weight and h) Seedlings of A. thaliana recovered after the period of water deprivation.
